# Factors associated with depression and anxiety among mental healthcare practitioners

**DOI:** 10.4102/jcmsa.v3i1.120

**Published:** 2025-02-21

**Authors:** Cheval Murugas, Carla Kotzé

**Affiliations:** 1 Department of Psychiatry, Faculty of Health Sciences, University of Pretoria, Pretoria, South Africa; 2 Weskoppies Psychiatric Hospital, Pretoria, South Africa

**Keywords:** psychiatry, mental health, depression, anxiety, mental healthcare workers

## Abstract

**Background:**

Anxiety and depression constitute a significant global mental health burden. The extant literature indicates elevated rates of anxiety and depression among mental healthcare practitioners; however, this phenomenon remains understudied within this particular population.

**Methods:**

A cross-sectional study was conducted using self-administered questionnaires. Participants voluntarily completed an anonymous socio-demographic questionnaire, the General Anxiety Disorder-7 scale and the Patient Health Questionnaire-9.

**Results:**

The study included 255 participants from various occupational categories within mental health. High levels of anxiety (*N* = 56; 22%) and depression (*N* = 42; 16.5%) were found among mental healthcare practitioners. Participants with a prior history of anxiety and/or depression (21.2%) demonstrated a significant association with anxiety and depression at the time of completion of the questionnaire (*p* = 0.000).

**Conclusion:**

Mental health practitioners with a previous history of anxiety and depression were at a substantially higher risk of experiencing current symptoms, highlighting the need for workplace support mechanisms tailored to this vulnerable group.

**Contribution:**

This research identified a specific group of mental health practitioners at elevated risk for anxiety and depression that warrants targeted interventions through occupational assistance initiatives and preventative strategies.

## Introduction

Globally, mental disorders affected one in eight individuals in 2019, with anxiety and depression being the most prevalent. Worldwide figures for that year indicated that 301 million people were living with an anxiety disorder and 280 million people were living with depression.^1,2^

A secondary analysis of health insurance data in Korea revealed significantly high rates of mental health issues among healthcare practitioners.^3^ The coronavirus disease 2019 (COVID-19) pandemic has added to the mental health burden among healthcare workers.^4,5^ Studies from various countries, including China, Singapore and European nations found increased rates of anxiety and depression among frontline healthcare workers.^6,7,8^ A cross-sectional survey in Ethiopia on psychosocial work conditions found that being female, experiencing high job demands and having low job control significantly contributed to healthcare workers’ psychological distress.^9^ Factors such as prior mental health challenges, living circumstances and marital status have been associated with anxiety and depression.^10,11,12,13,14,15^ A recent study conducted in KwaZulu-Natal, South Africa concluded that medical doctors working in resource-constrained training hospitals exhibit a high prevalence of anxiety and depressive symptoms.^16^

Healthcare practitioners working specifically in the field of mental health are also at risk for anxiety and depression.^17^ Research examining depressive symptoms in 2084 psychiatrists from North America revealed that 16% of the participants were experiencing depression at the time of assessment.^18^ Nurses working in the field of psychiatry experience high work-related stress which serves as a significant contributing factor to disorders such as anxiety and depression.^19,20^ A quantitative cross-sectional study examining 110 mental health nurses in Greece revealed significantly high rates of depression (52.7%) and anxiety (48.2%).^21^ Another investigation conducted at a large state psychiatric hospital in Nigeria showed that violence against mental health staff was highly prevalent and concluded that nursing staff were more frequently assaulted than doctors.^22^ A review examining burnout among mental healthcare professionals emphasised the necessity for further studies in this area and suggested that the psychological strain of providing care may increase these practitioners’ vulnerability to developing mental health issues. Mental healthcare practitioners are exposed to numerous occupational risks, including patients exhibiting violent or aggressive behaviour. Psychological reactions such as anxiety and depression are commonly reported by healthcare practitioners who were victims of workplace violence.^23^

Given the prevalence of anxiety and depression among healthcare workers and the role of mental health professionals, this subject warrants further and more comprehensive research. There is a paucity of research on this topic in South Africa, particularly with a specific focus on those practising in mental healthcare. Because of the lack of available literature, our research encompassed all mental health practitioners, including occupational therapists, social workers, psychology interns, psychiatry registrars, professional nurses or enrolled nurses, psychologists and psychiatrists, with a focus on the proportion of practitioners working at a tertiary psychiatric hospital in South Africa with symptoms of depression and anxiety. Additionally, we investigated the sociodemographic and occupational factors associated with symptoms of depression and anxiety in this specific population.

## Research methods and design

### Study design and setting

This study was conducted at Weskoppies Psychiatric Hospital situated in Tshwane, Gauteng province, South Africa. This is a tertiary-level psychiatric hospital offering in- and outpatient care, treatment and rehabilitation for child and adolescent, adult, geriatric and forensic mental healthcare users. The hospital also offers education and training in mental health for medical students, nurses and various other mental health practitioners including clinical psychology interns and registrars in psychiatry. This study used a cross-sectional design that employed self-administered questionnaires completed by mental healthcare practitioners employed at the hospital.

### Study population and sampling strategy

The study population consisted of different mental healthcare practitioners, including psychiatrists, registrars training to become psychiatrists, clinical psychologists, psychology interns, occupational therapists, occupational therapy assistants, social workers, professional nurses, enrolled nurses and enrolled nursing assistants currently working at the hospital. The study was available to those who were 18 years or older, willing to participate, able to provide informed consent, and able to read and comprehend the English language. Participants excluded were those who were not dealing directly with mental healthcare users. Non-probability, purposive sampling was used. The heads of each department at Weskoppies Hospital were informed about the research. The questionnaires were made available in all the relevant sections of the hospital for participants to complete during a 4-month period from April 2022 until July 2022. The information leaflet and informed consent form were distributed together with the questionnaires for participants to complete at a time that would not interfere with their service delivery. Sealed boxes were made available in each section for participants to submit the completed documents at a convenient time. We then checked these boxes on a weekly basis to collect completed questionnaires.

### Data collection

Participants provided written consent and then completed a demographic form and two validated screening instruments: the General Anxiety Disorder 7 scale (GAD-7) and the Patient Health Questionnaire 9 (PHQ-9).

The GAD-7 is a tool used to measure the severity of generalised anxiety disorder, and it also has adequate sensitivity and specificity for other anxiety disorders. The GAD-7 is composed of seven questions that rate the frequency of anxiety symptoms categorised as ‘not at all’ with a score of 1, ‘several days’ with a score of 2 and ‘nearly every day’ with a score of 3 which contributes to the index of severity. The GAD-7 has a specificity of 82% and a sensitivity of 89% for generalised anxiety disorders when using a cut-off score of 10 for those with moderate to severe anxiety.^24^

The PHQ-9 was used to measure symptoms of depression. It contains nine questions that screen for symptoms of depression. Responses to questions with ‘not at all’ gets a score of 0, ‘several days’ gets a score of 1, ‘more than half the days’ gets a score of 2 and ‘nearly every day’ results in a score of 3. The total score ranges from 0 to 27. A cut-off score of 10 or higher has a specificity of 88% and a sensitivity of 88% for major depression or depression of moderate to severe degree.^25^ Demographic data collected included age, self-identified gender, living circumstances, job status and marital status.

### Data analysis

All data collected was captured in an Excel spreadsheet. Statistics were presented with frequencies and percentages for symptoms of anxiety and depression among the different categories of mental healthcare practitioners in Weskoppies Hospital. Demographic data were presented as percentages for categorical variables. A total score for GAD-7 and PHQ-9 was given for different groupings of mental healthcare practitioners. The total scores for GAD-7 and PHQ-9 were categorised into less than 10 and greater than 10. The Chi-square test was used to determine associations between the categorised GAD-7 and PHQ-9 and other categorical demographic variables. *p*-values of less than 0.05 will be considered statistically significant.

### Ethical considerations

Ethical clearance to conduct this study was obtained from the University of Pretoria Faculty of Health Sciences Research Ethics Committee (reference no.: 25/2022).

## Results

The descriptive data for the demographic and occupational details and associations with anxiety and depression using a cut-off score of ≥ 10 for the GAD-7 and PHQ-9 are summarised in Table 1.

**TABLE 1:** Demographic and occupational details and associations with anxiety and depression (*N* = 255).

Categories	Totals		Association with anxiety (≥ 10)			Association with depression (≥ 10)		
	*n*	%	*p*	*n*	%	*p*	*n*	%
Age range (mean age 39.8-years):	-	-	0.443	-	-	0.297	-	-
• < 30	55	21.6	-	14	5.5	-	15	5.9
• 30–49	149	58.4	-	34	13.3	-	21	8.2
• > 50	51	20	-	8	3.3	-	6	2.4
Self-identified gender:	-	-	0.436	-	-	0.791	-	-
• Female	179	70.2	-	43	16.9	-	31	12.2
• Male	75	29.4	-	13	5.1	-	11	4.3
• Undisclosed	1	0.4	-	0	0	-	0	0
Marital status:	-	-	0.758	-	-	0.519	-	-
• Single	126	49.4	-	30	11.8	-	26	23.5
• Married/partnered	111	43.5	-	24	9.4	-	14	5.5
• Other (divorced, widowed or undisclosed)	18	7.1	-	2	0.8	-	2	0.8
Living status:	-	-	0.283	-	-	0.534	-	-
• Living alone	69	27.1	-	12	4.7	-	13	5.1
• Living with others (mostly spouse/children)	186	72.9	-	44	17.3	-	29	11.4
Occupational categories:	-	-	0.871	-	-	0.156	-	-
• Psychiatrists and registrars	25	9.8	-	5	2	-	1	0.4
• All categories of nurses	191	74.9	-	43	16.8	-	32	13.7
• Social workers	8	3.1	-	2	0.8	-	1	0.4
• Occupational therapists and assistants	14	5.5	-	3	1.2	-	4	1.6
• Psychologists and interns	17	6.7	-	3	1.2	-	4	1.6

High rates of anxiety (22%) and depression (16.5%) were found among mental healthcare practitioners, but there were no statistically significant findings between any of the demographic or occupational categories and the presence of anxiety or depression according to the GAD-7 or PHQ-9 with a cut-off score of ≥ 10. The mental health history and current anxiety and depression findings are summarised in Table 2.

**TABLE 2:** History of anxiety and/or depression and current symptoms of anxiety and depression.

Variable	Association with current anxiety (≥ 10)			Association with current depression (≥ 10)		
	*p*	*n*	%	*p*	*n*	%
History of anxiety and/or depression (*N* = 54; 21.2%)	-	56	22	-	42	16.5
Pearson Chi-square	0.000	-	-	0.000	-	-

The only statistically significant association identified was between current experiences of anxiety and depression at the time of the study and prior history of depression and/or anxiety (*p* = 0.000).

## Discussion

In keeping with what has been reported in other studies, there were high rates of anxiety (20%) and depression (21%) among mental healthcare practitioners in the current study.^3,16,26^ In this study there were no significant correlations found between the sociodemographic factors that were captured, including gender, occupation, marital and living status with anxiety and depression. This is different from what has previously been reported where being single, female and working as a nurse has been reported as risks for mental health-related problems among mental healthcare practitioners.^9,21,22,27^

The main finding of our study was that a previous history of anxiety and depression is significantly associated with the presence of current symptoms. The severity of these symptoms was associated with a prior history of depression/anxiety or both. This highlights the importance of and how a history of previous mental health problems can predict future mental health challenges and has important implications for workplace interventions. In the occupational setting, this group with a prior history of anxiety and depression should be considered as particularly vulnerable to experiencing anxiety and depression and should be a focus of interventions targeted at the prevention of recurrence. A possible explanation for the high rates of recurrence of anxiety and depression in this group who previously experienced mental health issues can be the stress and demand inherent to the mental healthcare profession. Stress plays a crucial role in increasing the risk of anxiety and depression and interventions should focus on mitigating the negative effects of chronic work-related stress.^28^

Furthermore, the organisational culture should promote staff well-being and address the stigma associated with mental health problems within the profession. Stigma may hinder help-seeking and adequate treatment is essential to prevent recurrence of persistence of symptoms. Addressing the structural aspects of stigma that might be lodged in the health system can have a positive impact on staff and potentially improve mental health outcomes. Early identification, continuous support and destigmatisation efforts for staff with a history of anxiety and depression should be prioritised to mitigate the recurrence of symptoms.^29^

Limitations of the study include the cross-sectional nature and not following up at-risk individuals over time. The survey did not include any questions about treatments that the participants might have been using or consider other possible contributing factors, such as medical conditions that might have influenced the reported anxiety and depression symptoms. Factors such as workload and personal coping mechanisms were not considered. The study was restricted to Weskoppies Psychiatric hospital.

## Conclusion

Our study reveals a significant association between a history of anxiety and depression and the recurrence of symptoms among mental healthcare practitioners working in psychiatric hospitals. The demanding work environment may contribute to symptom recurrence or persistence. To build resilience, institutions should proactively support staff with a history of mental health issues. Future research should focus on specific risk factors for depression and anxiety in mental healthcare practitioners to enhance targeted interventions. Prioritising mental health support and reducing stigma can better safeguard the well-being of those caring for others.
